# A dry polymer nanocomposite transcutaneous electrode for functional electrical stimulation

**DOI:** 10.1186/s12938-024-01200-8

**Published:** 2024-01-26

**Authors:** Melissa Marquez-Chin, Zia Saadatnia, Yu-Chen Sun, Hani E. Naguib, Milos R. Popovic

**Affiliations:** 1 KITE – Toronto Rehabilitation Institute, University Health Network, Toronto, ON Canada; 2 Institute of Biomedical Engineering, University of Toronto, Toronto, ON Canada; 3 Department of Mechanical & Industrial Engineering, University of Toronto, Toronto, ON Canada; 4 Department of Mechanical and Manufacturing Engineering, Ontario Tech University, Oshawa, ON Canada

**Keywords:** Dry electrode, Functional electrical stimulation, Torque, Comfort

## Abstract

**Background:**

Functional electrical stimulation (FES) can be used in rehabilitation to aid or improve function in people with paralysis. In clinical settings, it is common practice to use transcutaneous electrodes to apply the electrical stimulation, since they are non-invasive, and can be easily applied and repositioned as necessary. However, the current electrode options available for transcutaneous FES are limited and can have practical disadvantages, such as the need for a wet interface with the skin for better comfort and performance. Hence, we were motivated to develop a dry stimulation electrode which could perform equivalently or better than existing commercially available options.

**Methods:**

We manufactured a thin-film dry polymer nanocomposite electrode, characterized it, and tested its performance for stimulation purposes with thirteen healthy individuals. We compared its functionality in terms of stimulation-induced muscle torque and comfort level against two other types of transcutaneous electrodes: self-adhesive hydrogel and carbon rubber. Each electrode type was also tested using three different stimulators and different intensity levels of stimulation.

**Results:**

We found the proposed dry polymer nanocomposite electrode to be functional for stimulation, as there was no statistically significant difference between its performance to the other standard electrodes. Namely, the proposed dry electrode had comparable muscle torque generated and comfort level as the self-adhesive hydrogel and carbon rubber electrodes. From all combinations of electrode type and stimulators tested, the dry polymer nanocomposite electrode with the MyndSearch stimulator had the most comfortable average rating.

**Conclusions:**

The dry polymer nanocomposite electrode is a durable and flexible alternative to existing self-adhesive hydrogel and carbon rubber electrodes, which can be used without the addition of a wet interfacing agent (i.e., water or gel) to perform as well as the current electrodes used for stimulation purposes.

**Supplementary Information:**

The online version contains supplementary material available at 10.1186/s12938-024-01200-8.

## Background

Functional electrical stimulation (FES) consists on the application of low energy electrical pulses to the peripheral nerves to activate the neuromuscular junction and induce muscle contractions resulting in functional movement. FES can be used in rehabilitation to assist or restore movement in people with paralysis [1–6]. To deliver stimulation, the minimum setup requirements are a stimulator and at least a pair of electrodes.

The stimulator is the device which provides the electrical pulses. The pulses can be of different shapes, widths, and amplitudes, and can be delivered at specific frequencies. The electrodes are the interface between the stimulator and the body. They can be categorized into invasive or non-invasive depending on whether they are placed directly on the nerve, through the skin, or on the surface of the skin. Invasive electrodes require a surgical procedure for implantation and deliver stimulation directly to a specific nerve or close to a motor neuron. Non-invasive electrodes are placed on the skin (i.e., transcutaneous) and the electrical impulses are delivered through the skin’s surface [1, 2]. Within the transcutaneous electrode category, the current standard for FES are self-adhesive hydrogel electrodes. These are multi-layered electrodes with an adhesive hydrogel in contact with the skin [7]. They are easy to use but tend to lose their adhesive properties in the short-term which makes them not ideal for reuse.

Carbon rubber electrodes are another type of transcutaneous electrode made from carbon and silicone. These are non-adhesive electrodes which usually require an added layer of gel or water at the skin interface. They have a high impedance but can be less practical to use, since they may require repeated wetting, and the use of additional straps, tape, or wraps to hold them in place [7].

Textile-based electrodes, which can be integrated into a garment, are another type of transcutaneous electrode which has been more recently explored [7–10]. Most use silver or carbon-based coatings on different substrates as the conductive material. However, these electrodes usually require a wet interface (i.e., either gel or water) to have a proper skin–electrode interface and less discomfort [9, 11]. The application of water to the electrodes has been identified by users as one of the major disadvantages that require improvement for garments using these electrodes to be used in practical situations [12].

The level of comfort may affect users’ tolerance and adherence to stimulation, so a higher comfort level could potentially enable the use of FES for longer periods of time or to produce stronger muscle contractions. Since current transcutaneous electrode options require the use of a wet interface to improve performance and increase comfort, the constant addition of gel or water can become impractical and uncomfortable for users. In this article, we present the use of a completely dry transcutaneous electrode for stimulation purposes which does not require a wet interface to operate effectively. The electrode is as a thin film made from a polymer nanocomposite blend (PVDF + CNT) which is durable, flexible, and has a smooth non-adhesive surface. We compared this electrode’s performance to two standard transcutaneous electrodes in combination with three different stimulators to verify its viability for stimulation and to compare which electrode, stimulator, or combination could generate the strongest muscle contraction with the most comfort.

## Results

### Thermal, mechanical, and electrical characterization

**Fig. 1:**
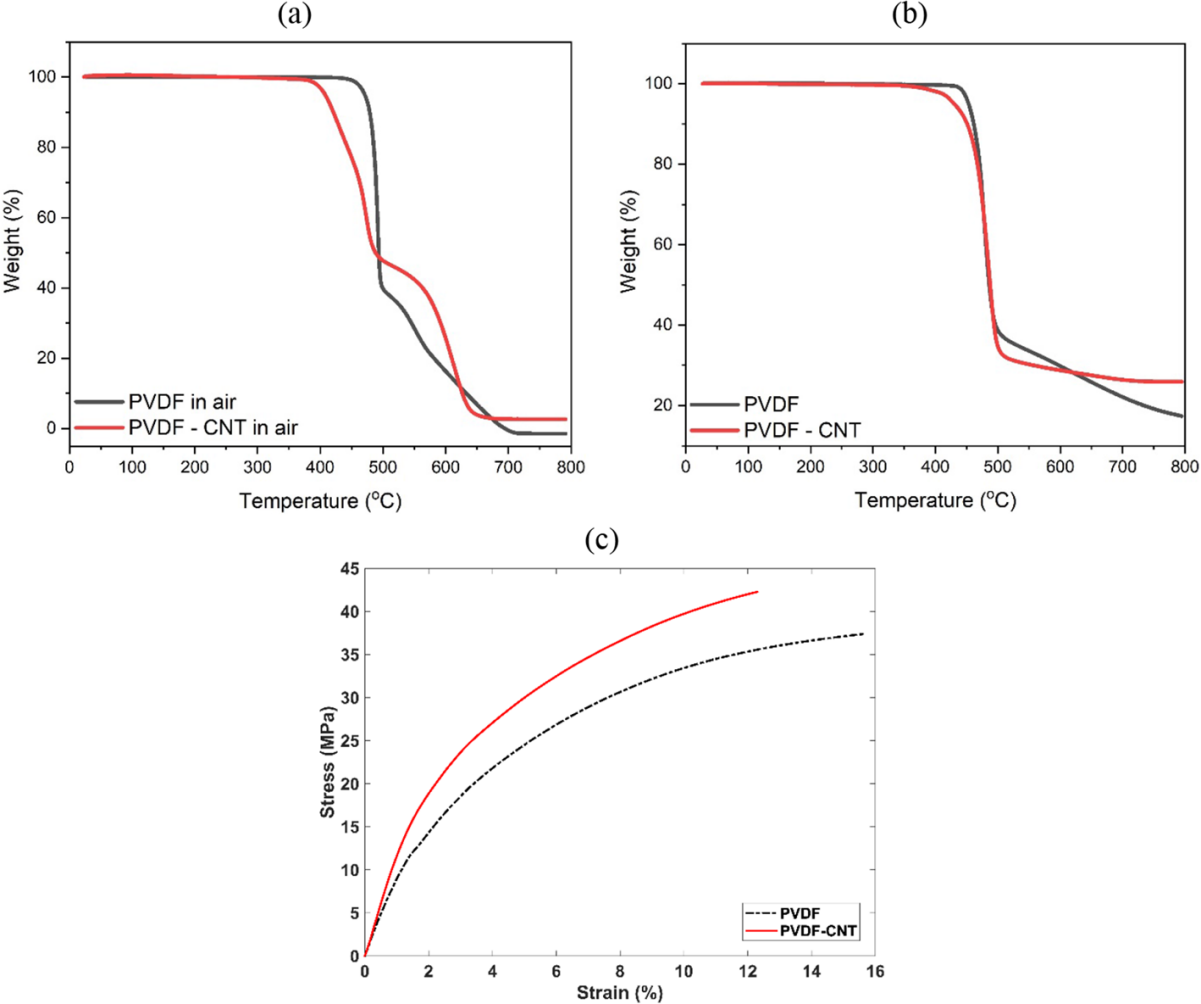
TGA curves under two environments: **a** air and **b** nitrogen. **c** Tensile stress–strain curves of the dry electrode polymer nanocomposite and pristine polymer

As the dry reusable electrodes are ultimately targeted toward wearable applications, they will most likely be exposed to high temperature, such as a clothes dryer or potentially ironing. Therefore, it is important to understand the material’s thermal stability behaviour at elevated temperatures. Such thermal behavior of the polymer nanocomposite and pristine polymer, using Thermogravimetric Analyses (TGA) under air environment, are shown in Fig. 1(a). The initial degradation temperatures of the PVDF samples were around 436 ± 2 °C and 383 ± 2 °C for the pristine polymer and CNT nanocomposite, respectively. It can be observed that both samples are thermally stable up to 350 °C, which imply the electrode is safe for a day-to-day usage from the respective of thermal degradation. Samples showed relatively high initial degradation temperatures and the slight difference could be attributed to the dispersion of the CNT in the polymer matrix, similar to the results reported by [13]. The reduction in the onset temperature is likely due to the CNT acting as a defect between the polymer matrix and the interface sites initiating the degradation, therefore reducing the initial onset degradation temperature [13]. Above 700 °C, all samples were fully degraded as the final weight is close to 0%. For comparison, the thermal stability results from standard TGA test under nitrogen environment is also shown in Fig. 1(b) where the initial degradation temperatures of around 445 ± 2 °C and 429 ± 2 °C were found for the pristine polymer and the polymer nanocomposite, respectively.

After mechanical testing, the stress–strain curves for the dry electrode and pristine polymer samples are presented in Fig. 1(c). As expected, the addition of a small percentage of CNT, which owns higher stiffness and strength, into the polymer matrix resulted in the increase on the elastic modulus and the yield strength of the polymer composite film compared to the pristine polymer film. The stiffness represented by the elastic modulus was found to be around 1.19 ± 0.04 GPa and 0.92 ± 0.05 GPa for the polymer composite film and the pristine polymer film, respectively. The strength of the samples presented by the yield strength was obtained around 16.3 ± 0.1 MPa and 12.7 ± 0.1 MPa for the polymer composite and pristine polymer, respectively. The elongation of the pristine polymer at lower stresses is relatively higher than the polymer composite due to higher stiffness of the polymer composite. Overall, the increased stiffness and strength of the dry electrode film material due to the small percentage addition of the CNT remained within reasonable ranges compared to the thermoplastic PVDF polymer allowing for its application for electrical stimulation purposes with suitable flexibility and durability.

**Fig. 2:**
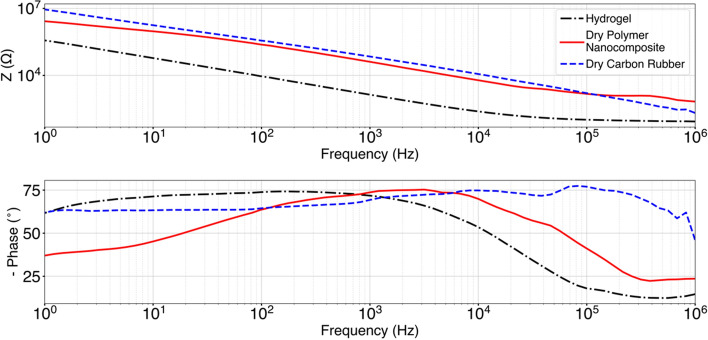
Impedance magnitude and phase response of the hydrogel, dry polymer nanocomposite, and dry carbon rubber electrodes on a person’s forearm

From the electrical testing, the average impedance of the hydrogel electrodes for the frequency range tested was 36.06 ± 77.70 kΩ, for the dry polymer nanocomposite samples the average impedance was 401.19 ± 664.63 kΩ, and for the dry carbon rubber electrodes the average impedance was 970.51 ± 1933.12 kΩ. The impedance and phase results are shown in Fig. 2. In addition, the average surface resistivity of the samples measured was 1.46 ± 2.06 MΩ for the hydrogel electrode, 261.66 ± 85.42 Ω for the dry polymer nanocomposite electrode, and 628.33 ± 198.74 kΩ for the dry carbon rubber electrode.

### Stimulation intensities

**Fig. 3:**
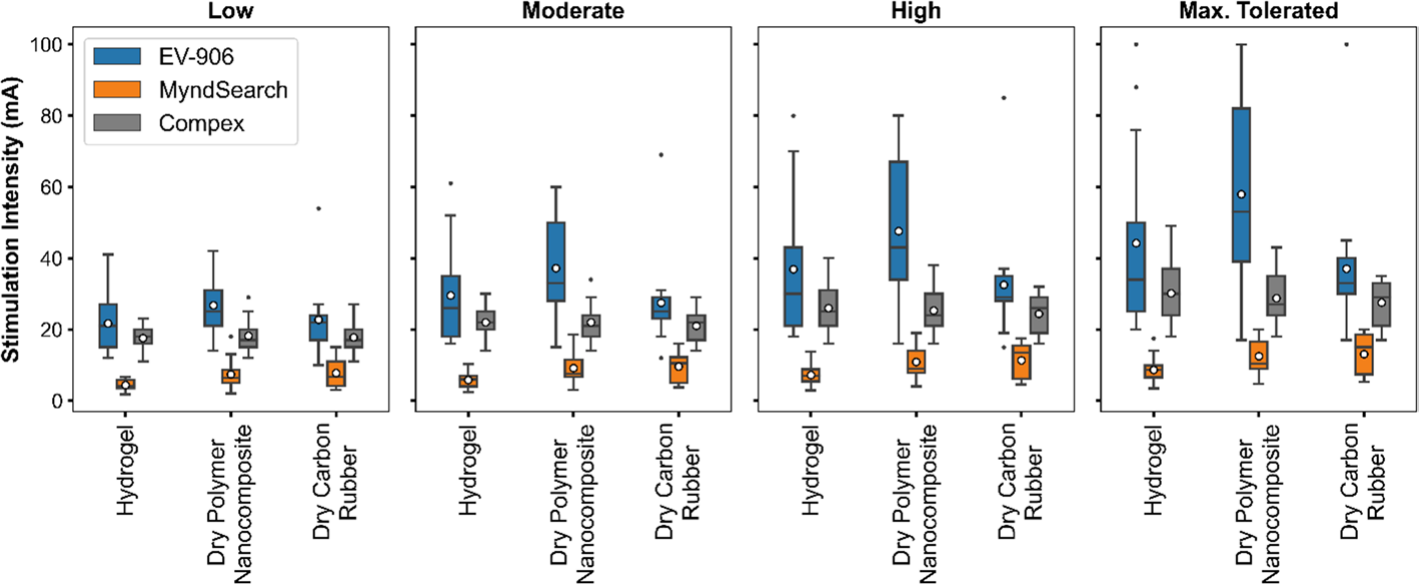
Average stimulation intensities used for each electrode and stimulator type used. In each boxplot, the horizontal line indicates the median and the circle the mean

**Fig. 4:**
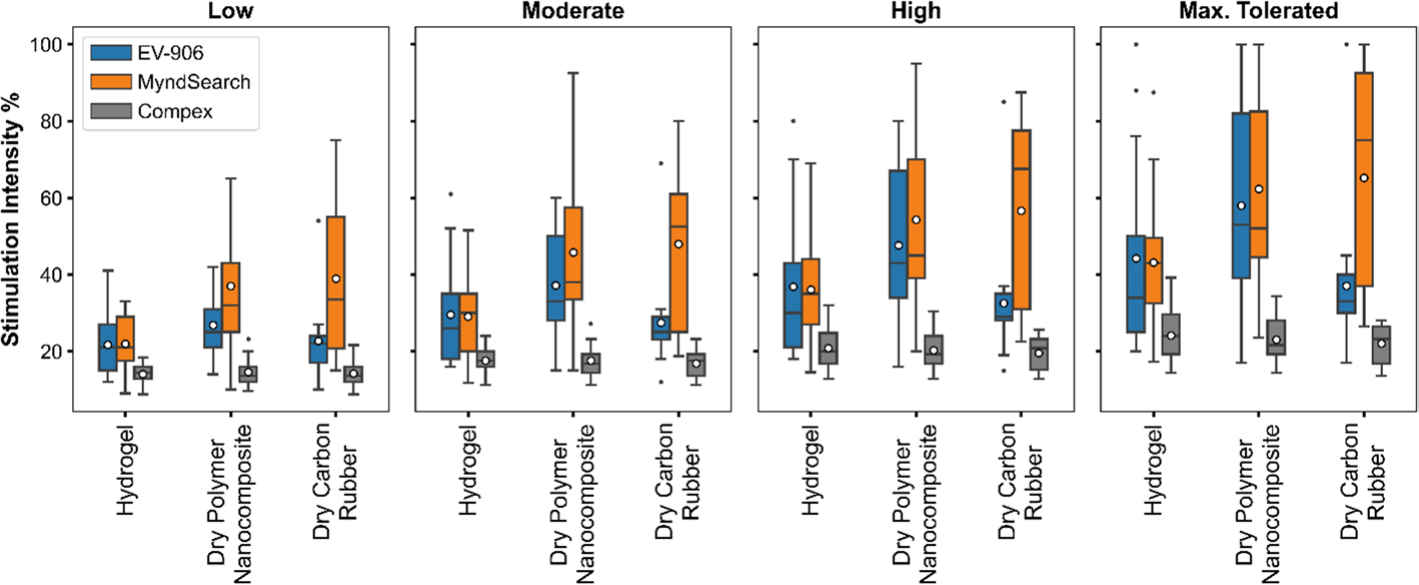
Average percentage of stimulation intensities used based on each stimulator’s maximum possible output. In each boxplot, the horizontal line indicates the median and the circle the mean

Since we used different stimulation intensities for each combination of electrode–stimulator, we compared the average current amplitudes for all individuals at each intensity level, as shown in Fig. 3. Overall, we could use higher intensities with the dry polymer nanocomposite electrode with the EV-906 and the MyndSearch stimulators. For the Compex Motion, all electrode types used similar intensities on average. However, it should be noted that each stimulator had an established pulse intensity limit, and four individuals reached said limits with some electrode–stimulator combinations. One participant reached the maximum output from the MyndSearch stimulator with the dry polymer nanocomposite electrode only, one participant reached the maximum output of the EV-906 stimulator and the MyndSearch stimulator both with the dry polymer nanocomposite electrode, one participant reached the maximum output of the EV-906 stimulator with the hydrogel, the dry polymer nanocomposite, and the carbon rubber electrodes, and one participant reached the maximum MyndSearch stimulator output with the carbon rubber electrodes. The EV-906 device limit was 100 mA, the MyndSearch limit was 20 mA, and the Compex Motion limit was 125 mA. If we expressed each intensity as a percentage of the maximum possible output of each stimulator, as shown in Fig. 4, the dry polymer nanocomposite electrode and the dry carbon rubber allowed using higher intensities in combination with the MyndSearch given the device’s limits.

### Perceived comfort ratings

**Fig. 5:**
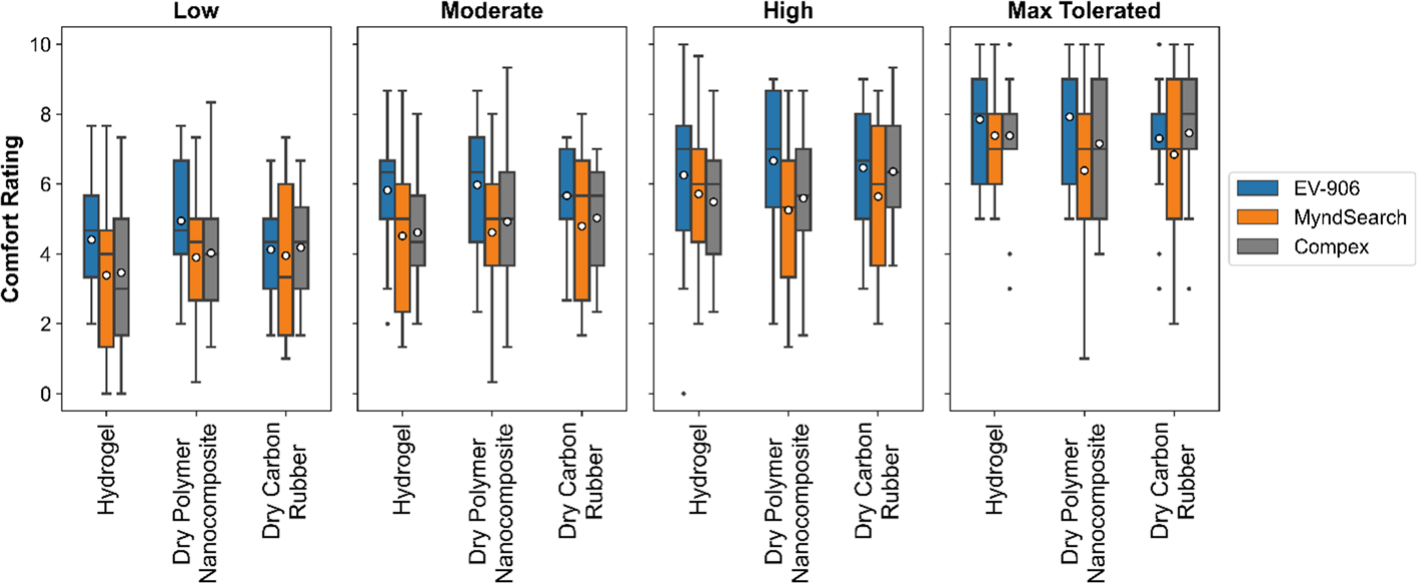
Average comfort ratings reported, where 0 is the most comfortable and 10 the most uncomfortable, for each intensity level

The average perceived comfort rating between all individuals for each electrode–stimulator combination is shown in Fig. 5. Based on the averages from the visual analog scale, throughout all intensity levels, the MyndSearch stimulator was the most comfortable compared to the other two stimulators. At low intensities the MyndSearch with the hydrogel electrode was the most comfortable combination with a total average comfort rating of 3.38 ± 2.27. At moderate intensities, the MyndSearch with the hydrogel electrode was the most comfortable combination with a total average comfort rating of 4.51 ± 2.42. At high intensities, the MyndSearch with the dry polymer nanocomposite electrode was the most comfortable combination with a total average comfort rating of 5.26 ± 2.28. At the maximum tolerated intensity level, the most comfortable combination was the dry polymer nanocomposite electrodes with the MyndSearch stimulator with a total average comfort rating of 6.38 ± 2.50. Each individual’s comfort rating for each combination can also be found in Additional file 1: Fig. S1. After doing a statistical analysis, we found no statistically significant difference (*p* < 0.05) in the comfort ratings between the three electrode types used, or the three different stimulators used. The *p* values for each electrode or stimulator tested can be found in Table 1.

**Table 1:** Statistical analysis *p* values for comfort ratings

	Electrode	Low intensity	Moderate intensity	High intensity	Maximum tolerated intensity
	Stimulator				
Difference between 3 stimulators used	Hydrogel	*p *= 0.278	*p* = 0.156	*p* = 0.449	*p* = 0.766
	Dry polymer nanocomposite	*p* = 0.378	*p* = 0.234	*p* = 0.187	*p* = 0.210
	Dry carbon Rubber	*p* = 0.951	*p* = 0.639	*p* = 0.636	*p* = 0.903
Difference between 3 electrodes types used	EV-906	*p* = 0.466	*p* = 0.734	*p* = 0.912	*p* = 0.671
	MyndSearch	*p* = 0.788	*p* = 0.872	*p* = 0.778	*p* = 0.677
	Compex Motion	*p* = 0.637	*p* = 0.751	*p* = 0.501	*p* = 0.938

**Fig. 6:**
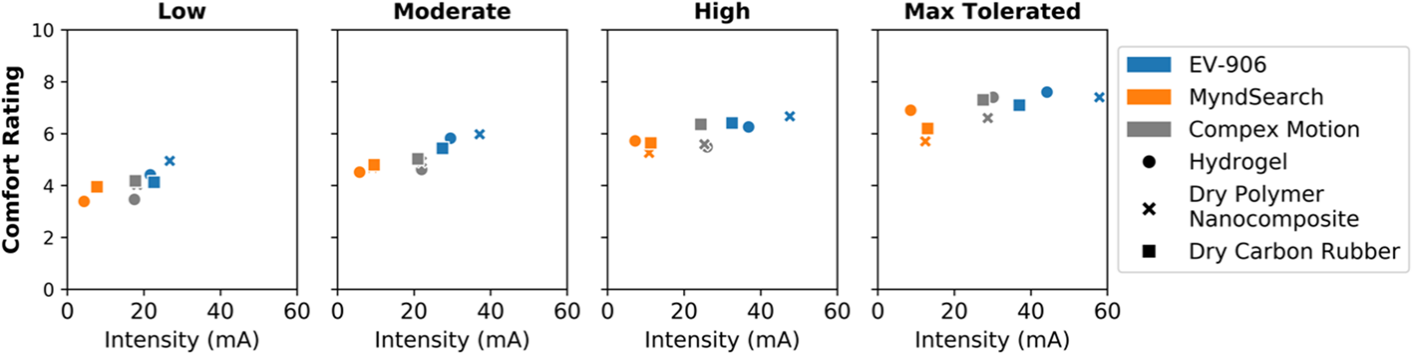
Average comfort ratings vs. intensity of stimulation used per electrode–stimulator combination

A comparison between the intensity of stimulation applied and the comfort level can also be seen in Fig. 6. The EV-906 stimulator had the biggest differences between intensities by electrode type used, but the comfort level was very similar between them. The MyndSearch stimulator required higher intensities with the dry electrodes than with the hydrogel, but both the dry polymer nanocomposite electrode and the dry carbon rubber electrode were more comfortable at the maximum tolerated intensity level. The Compex Motion stimulator used similar intensities for all electrode types, but the carbon rubber electrode was the most uncomfortable at all levels, and the dry polymer nanocomposite was more comfortable at the maximum tolerated level.

### Stimulation-induced muscle torque

**Fig. 7:**
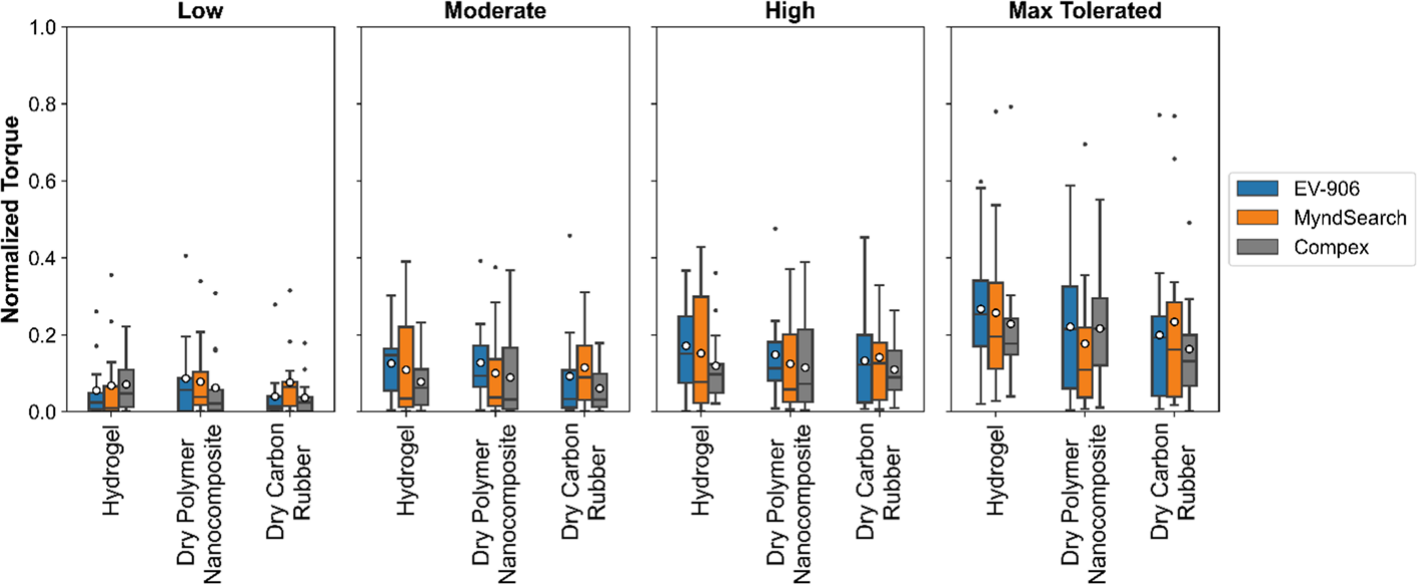
Average normalized muscle torque generated during different stimulation intensities

From the stimulation-induced torque values recorded, we used the average steady-state torque of each individual to do the comparison. The steady-state torque was defined as the average torque within the last second of stimulation at the intensity level being tested. The average of the three maximum voluntary contractions was used to normalize the torque for each individual. The stimulation-induced torques are presented in Fig. 7. At the low intensity level, the dry polymer nanocomposite electrode with the EV-906 stimulator generated the strongest contractions on average at 0.086 ± 0.116. At moderate intensity, the dry polymer nanocomposite electrode with the EV-906 stimulator generated the strongest contractions on average at 0.127 ± 0.105. At high intensity, the hydrogel electrode with the EV-906 stimulator generated the strongest contractions at 0.171 ± 0.121. At the maximum tolerable intensity, the hydrogel electrode with the EV-906 stimulator generated the strongest contractions at 0.267 ± 0.181. Each individual participant’s generated torque for each combination can be seen in Additional file 1: Fig. S2. After doing a statistical analysis to see if there was a significant difference in generated torque depending on the electrode type or stimulator used, we did not find any statistically significant difference (*p* < 0.05), as shown on the *p* values in Table 2.

**Table 2:** Statistical analysis *p* values for normalized generated torque

	Electrode	Low intensity	Moderate intensity	High intensity	Maximum tolerated intensity
	Stimulator				
Difference between 3 stimulators used	Hydrogel	*p* = 0.419	*p* = 0.491	*p* = 0.634	*p* = 0.711
	Dry polymer nanocomposite	*p* = 0.699	*p* = 0.258	*p* = 0.414	*p* = 0.526
	Dry carbon Rubber	*p* = 0.141	*p* = 0.309	*p* = 0.825	*p* = 0.816
Difference between 3 electrodes types used	EV-906	*p* = 0.649	*p* = 0.329	*p* = 0.673	*p* = 0.370
	MyndSearch	*p* = 0.613	*p* = 0.344	*p* = 0.550	*p* = 0.813
	Compex Motion	*p* = 0.381	*p* = 0.806	*p* = 0.848	*p* = 0.423

**Fig. 8:**
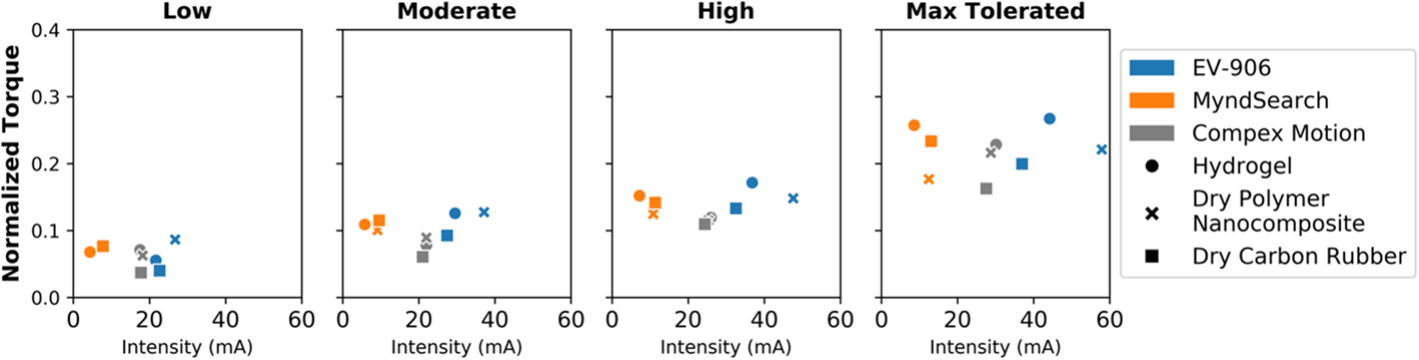
Average normalized generated torque vs. intensity of stimulation used per electrode–stimulator combination

Comparing the intensity of stimulation applied to the average amount of torque generated, shown in Fig. 8, the hydrogel electrodes produced the highest amount of torque with lower intensities than the dry electrode types at the high and maximum tolerated levels for the EV-906 and MyndSearch stimulators. While for the Compex Motion stimulator, slightly higher intensities could be used with the hydrogel electrodes, but the amount of torque was also higher.

### Reported sensations

**Fig. 9:**
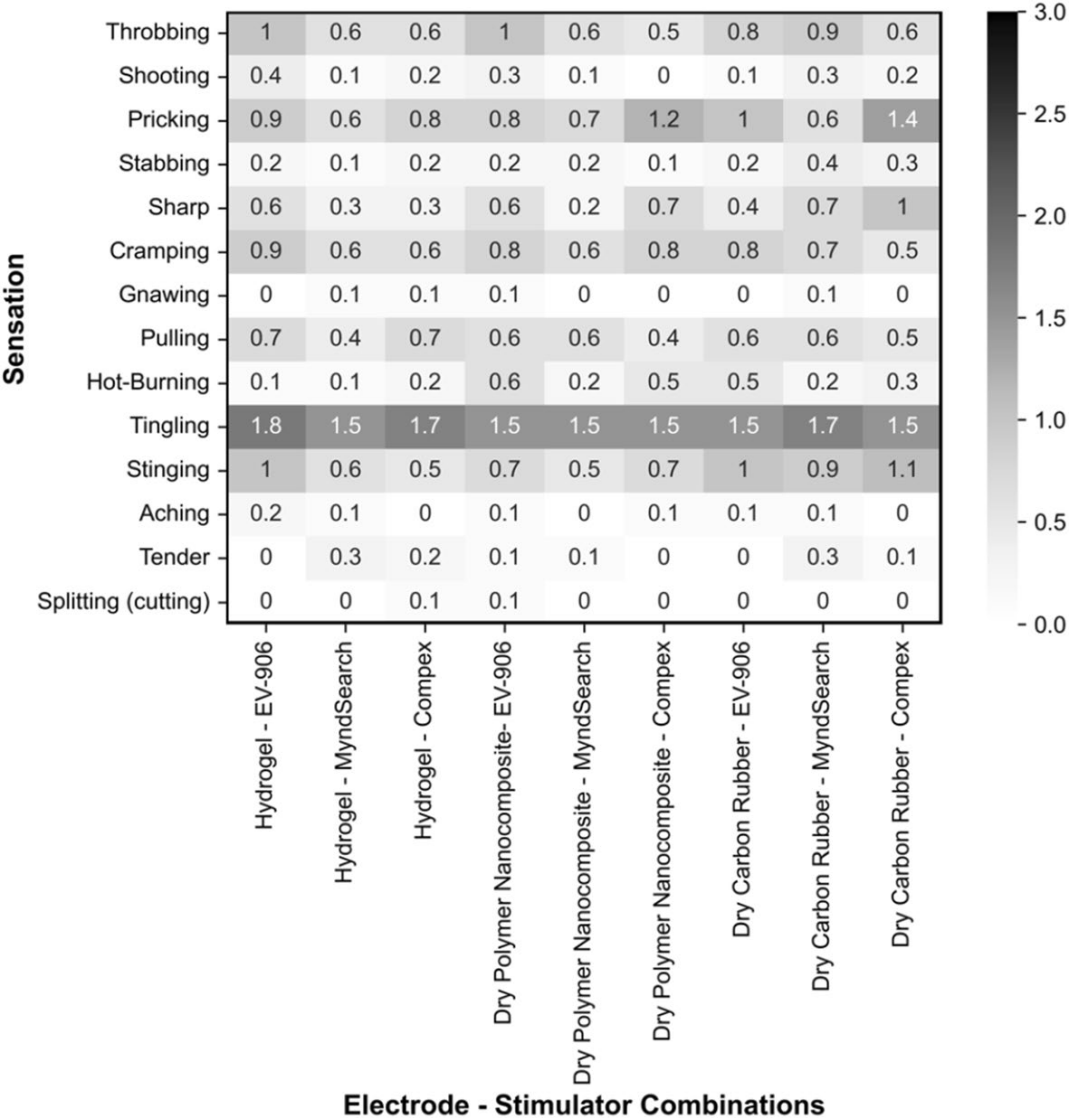
Average levels of sensations reported to be felt during stimulation

The average reported sensations from the given questionnaire are shown in Fig. 9. ‘Tingling’ was the most frequently reported sensation for all possible combinations and had very similar scores between 1.5 and 1.8 out of 3. Each individual participant’s reported sensations can also be found in Additional file 1: Fig. S3.

**Fig. 10:**
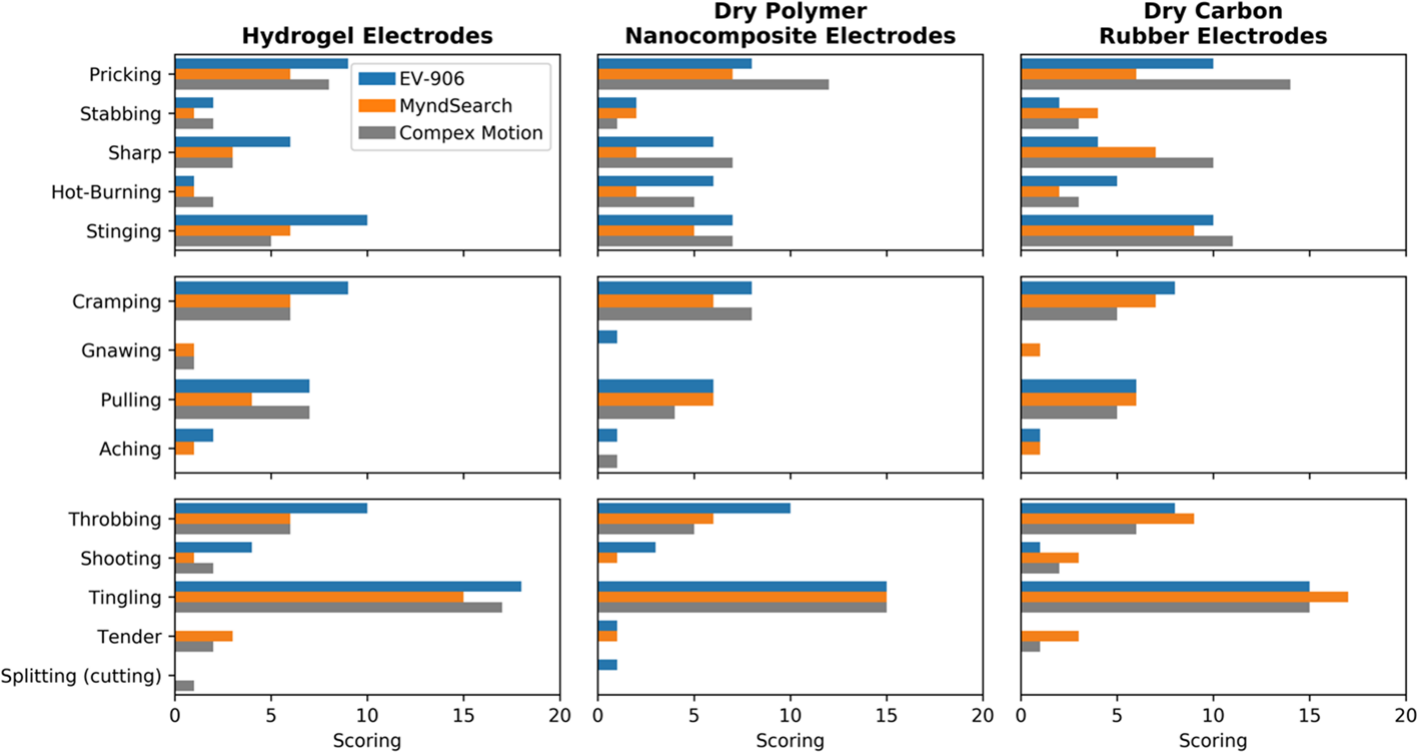
Combined scores of sensations per cutaneous (superficial), muscular (deep), and general categorization for each electrode type and stimulator used

The described sensations can be separated into three different categories as they are related to the level of nerve fibers activated [14–19]: cutaneous (superficial), muscular (deep), and general. Cutaneous sensations include ‘pricking’, ‘stabbing’, ‘sharp’, ‘hot-burning’, and ‘stinging’ [16–18]. Particularly, ‘pricking’ and ‘stinging’ have been associated with activation of Aδ fibers while ‘stabbing’, ‘sharp’, and ‘hot-burning’ have been associated with C fibers [15, 19]. Muscular sensations include ‘cramping’, ‘gnawing’, ‘pulling’, and ‘aching’[16–18]. These are all related to the activation of deep nociceptors and muscle pain. General sensations are ‘throbbing’, ‘shooting’, ‘tingling’, ‘tender,’ and ‘splitting (cutting)’, which are all associated with the stimulation of mechanoreceptors [14]. A combined added score of all sensations per category can be found in Fig. 10. All electrode types with all stimulators reported more cutaneous than deep sensations. In particular, the dry carbon rubber electrode with the Compex Motion had the most amounts of reported ‘pricking’, ‘sharp’ and ‘stinging’ sensations. Within the deep category, ‘cramping’ and ‘pulling’ were the most frequently reported sensations with all electrode and stimulator types.

## Discussion

The thermal testing showed that the dry polymer nanocomposite electrode is stable up to 350 °C which could enable these electrodes to be embedded into garments and potentially endure the temperatures of washing, drying, and ironing processes. After mechanical testing, we also demonstrated that the dry polymer nanocomposite electrode has a high tensile and yield strength which could also contribute to the durability of these electrodes in wearable applications. Compared to standard hydrogel electrodes, the dry polymer nanocomposite electrodes had a higher electrical impedance while on the skin, while the dry carbon rubber electrodes had the highest impedance. The dry polymer nanocomposite had a high impedance which could prevent high current concentrations on the skin and potentially reduce discomfort [7].

The use of stimulation with transcutaneous electrodes has the disadvantage of generally being more uncomfortable, since nociceptors on the skin and subcutaneous tissues are inevitably activated, as they are found between the electrodes and the muscle motor point [17, 18]. However, it has the advantage of being non-invasive and more accessible to use [6]. The level of comfort experienced by the user can be a factor in adherence to therapy and potentially stimulation contraction strength. Although some stimulators have been shown to be more comfortable than others, like the MyndSearch compared to the Compex Motion [20], we focused on the electrode type and its use in combination with different stimulators. Most transcutaneous electrode types require the addition of gel or water to improve the interface with skin and result in less discomfort [11]. However, in this work we demonstrated the use of a completely dry polymer nanocomposite electrode that performs as well as the standard self-adhesive hydrogel electrodes for stimulation purposes.

**Fig. 11:**
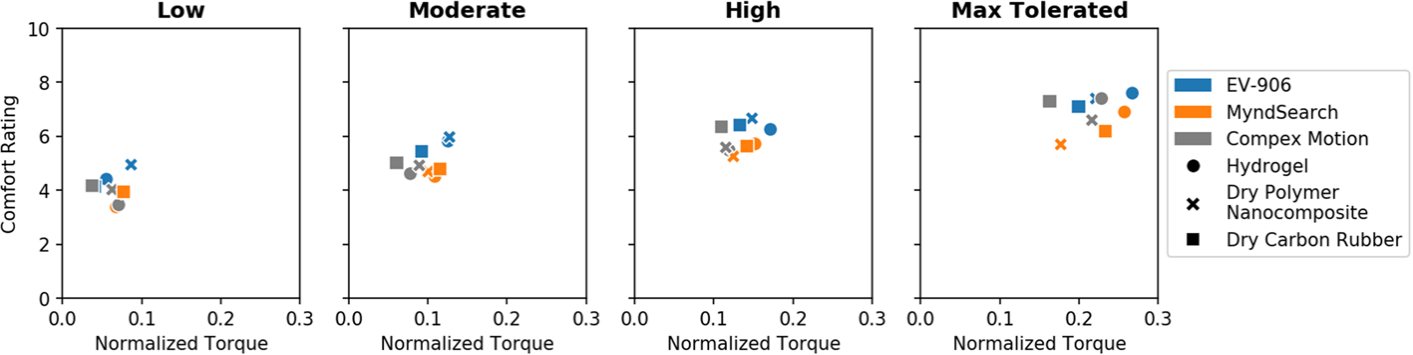
Average amount of normalized torque generated in relation to its perceived comfort level

When applying stimulation, the ideal would be to have the strongest muscle contraction (i.e., highest torque) with the most amount of comfort. To find which of the combinations of electrode types and stimulators used generated the strongest contractions with the least amount of discomfort, we also compared the average amount of torque in relation to its respective average comfort level as shown in Fig. 11. Although the differences were not statistically significant, the combination that produced the highest torque and was rated as the most comfortable at the maximum tolerated stimulation level, was the dry polymer nanocomposite electrode with the MyndSearch stimulator.

Throughout the comprehensive comparison between the three transcutaneous electrode types with three different stimulators, a summary of our findings would be:

- (1) Higher stimulation intensities could be used with the dry polymer nanocomposite electrode when combined with the MyndSearch or EV-906 stimulators, which could be related to less discomfort.
- (2) Numerical comfort ratings were not significantly different between electrode type or stimulator. However, the dry polymer nanocomposite electrode with the MyndSearch stimulator was overall rated as more comfortable than the other combinations at high and maximum tolerated intensities.
- (3) ‘Tingling’ was the most frequently descriptor for all combinations used, but for the dry carbon rubber electrode with the Compex Motion stimulator there were more cutaneous descriptors reported.
- (4) For the muscle torque generated, there was also no statistically significant difference between electrode type or stimulator used. Although slightly higher torques could be achieved with the hydrogel electrode than with the dry electrode alternatives using lower intensities. Plots showing a comparison between torque, comfort, and intensity can be found in Additional file 1: Fig. S4. Since there was no statistically significant difference in performance, this could imply that the new dry polymer nanocomposite electrode performs similarly to the current standard electrodes for stimulation purposes. However, the sample size should be considered as a limitation, and the varying individual differences in terms of preferred combination should also be taken into account.

Overall, the dry polymer nanocomposite electrode had a high yield strength and sufficient flexibility due to its thin film presentation, while also having a high impedance. It has a smooth surface and does not require the addition of gel or water to work as well as the current self-adhesive hydrogel electrodes available.

## Conclusion

We have presented a new completely dry electrode for electrical stimulation purposes that showed a similar performance to the current standard electrodes for stimulation, but without the need for a wet interface. It should be noted that even though there was no statistically significant difference, the average discomfort level was lower with the MyndSearch stimulator than with the other stimulators or electrodes used, while generating similar levels of torque. However, the exact reason why the dry electrode was more comfortable with this particular stimulator is unknown at the moment and could be a topic to be further investigated in the future. In future applications, this electrode could also be integrated into garments for stimulation, due to its reusable nature and smooth non-adhesive surface, and could potentially enable a more user-friendly form to deliver FES. When integrated into a garment, it could also facilitate the use of stimulation in settings outside the clinic.

## Materials and methods

### Materials for electrode fabrication

Multi-walled carbon nanotube (CNT-NC7000) was obtained from Nanocyl (Belgium) where the carbon nanotubes have the average diameter of 9.5 nm, average length of 1.5 µm, surface area of 25–300 m^2^/g, and volume resistivity of 10–4 Ω.cm on powder. Dimethylformamide (DMF) was purchased from Sigma-Aldrich (USA) and Polyvinylidene Fluoride (PVDF, Kynar 740) was supplied by Arkema (Canada). All materials were used without any further purification. Deionized (DI) water was used for all experiments.

### Electrode fabrication

To fabricate the dry polymer nanocomposite electrode, we used a standard solution casting process [21, 22]. First, a conductive filler, i.e., CNT (5 wt% of the polymer) was dispersed into a polymer solvent, i.e., DMF (93 wt% of the total solution) using a sonication process for 1 h at 60 Watts. Then, we added thermoplastic polymer pellets, i.e., PVDF, into the dispersion and mixed for four hours using a magnetic stirrer at 80ºC. After the polymer was fully dissolved, we casted the solution into a petri dish and dried it on a hot plate at 120 °C for 12 h. The dry PVDF–CNT blend was transferred into a compression molder, heated at 180 °C for 6 min, and then compressed into custom-made steel molds for three minutes. We obtained thin-film electrodes of approximately 100 µm thickness. After compression, the samples were cooled down in a water bath and removed from the mold.

### Thermal and mechanical characterization

To investigate the dry electrode material properties and the effect of the conductive nano filler (i.e., CNT), the thermal and mechanical properties of the polymer nanocomposite electrode material were examined and compared to the pristine PVDF polymer with no additives. The thermal degradation behavior of the polymer composite and pristine polymer were tested by thermal Thermogravimetric Analyses (TGA, Q50, TA Instruments, USA). The mechanical properties of the dry electrode and pristine polymer film samples were tested and compared with each other to show the mechanical stiffness and strength of the developed electrode. For this purpose, the tensile stress–strain behavior of three film samples was obtained by means of a Dynamic Mechanical Analyzer (DMA, Q800, TA Instruments, USA) using the force rate of 1 N/min.

### Electrical characterization

To determine the electrical properties of the three types of electrodes used, we measured the impedance of each electrode (hydrogel, dry polymer nanocomposite, and dry carbon rubber) using electrochemical impedance spectroscopy (EIS) with an electrochemical analyzer (Model CHI6054E, CH Instruments, USA). The experimental setup consisted of a three-electrode configuration with one sample connected to the working electrode, a second sample connected to the counter electrode, and a third sample connected to the reference electrode while placed on a single subject’s forearm (female, age 30). The parameters used were a sine wave with a peak amplitude of 0.01 V and a frequency range from 1 Hz to 1 MHz. For comparison, this procedure was performed on samples with a 5 × 5 cm square shape design. In addition, the average surface resistivity of the samples was measured using a digital multimeter (34401A Multimeter, Agilent, USA).

### Functional electrical stimulation (FES) testing

To test how efficient the new dry electrode was for FES purposes, we stimulated thirteen healthy individuals on their upper arm while measuring muscle torque and perceived comfort or discomfort levels using different electrode types and stimulator combinations. Among the subjects, there were five male and eight female participants whose demographics can be seen in Table 3. All participants were informed of the protocols to be used before any testing and signed an informed consent form which was approved by University Health Network’s Research Ethics Board (ID#21–5298).

**Table 3:** Participants demographics

Age (years)	Sex	Weight (kg)	Height (*m*)	Dominant hand
48	Male	96	1.82	Right
29	Female	65	1.65	Right
31	Female	50	1.63	Right
24	Female	59	1.70	Right
26	Female	63	1.72	Right
26	Female	50	1.50	Right
29	Female	50	1.57	Right
25	Male	77	1.80	Right
57	Male	95	1.85	Left
31	Male	75	1.73	Right
26	Male	75	1.78	Right
35	Female	75	1.60	Right
29	Female	51	1.60	Right

**Fig. 12:**
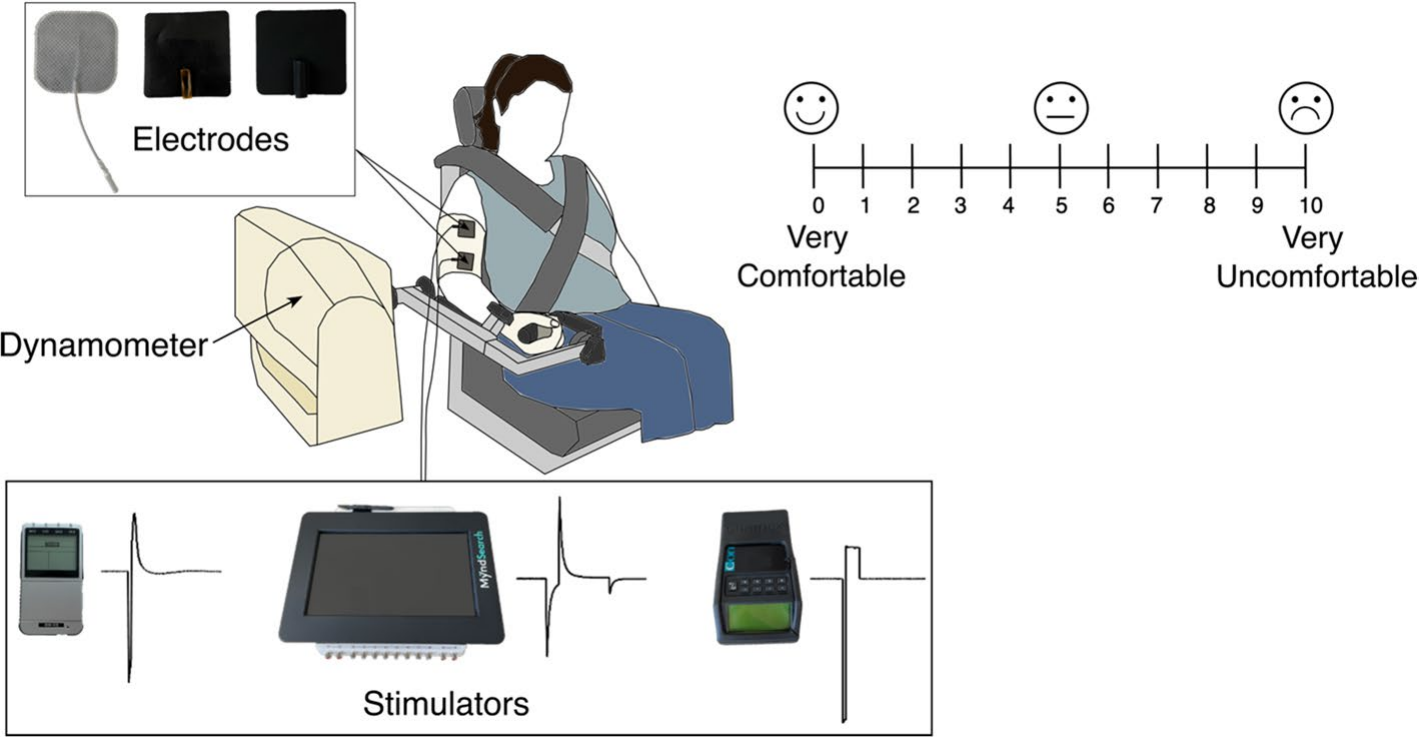
Experimental setup and visual analog comfort rating scale. Different electrode types used from left to right: hydrogel, dry polymer nanocomoposite, and carbon rubber. Stimulators used with their respective pulse shape from left to right: EV-906, MyndSearch, and Compex Motion

We used three different electrode types: standard self-adhesive hydrogel (ValuTrode 5 × 5 cm, Axelgaard Manufacturing, Denmark), carbon rubber without electrolyte (i.e., gel or water) added (5 × 5 cm, AMG Medical Inc, Canada), and our dry polymer nanocomposite electrode. We tested each electrode type with each of the three following stimulators: a portable battery-operated stimulator (EV-906, Everyway Medical Instruments, Taiwan), a MyndSearch stimulator (MyndTec Inc, Canada), and a Compex Motion stimulator (Compex SA, Switzerland). The three stimulator’s parameters were set at a pulse frequency of 40 Hz, pulse width of 300 µs, and pulse amplitude dependent on each participant. Each stimulator had a specific pulse shape shown on Fig. 12. The EV-906 stimulator generates voltage-regulated pulses, the MyndSearch generates stimulation pulses that are both voltage and current regulated (voltage regulation is controlled by the inner loop and current regulation is controlled by the outer loop of a closed-loop controller), and the Compex Motion generates current regulated stimulation pulses.

A pair of electrodes was placed on each subject’s right biceps brachii, with the anode placed on the proximal end of the muscle and the cathode on the distal end of the muscle belly. After the first electrode type was tested, we marked the outline of the electrode placement to position all other electrode types in the same area. The carbon rubber electrodes and the dry polymer nanocomposite electrodes were fixated to the skin using medical tape along the sides. All electrode types, including the self-adhesive hydrogel electrodes, were wrapped with self-adherent wrap to keep them in place. For each individual, we determined their sensory threshold (i.e., the amplitude when the person started to feel any sensation related to the stimulation), minimum contraction threshold (i.e., mCT, the amplitude when the person’s muscles started to twitch or contract), and maximum tolerated contraction threshold (i.e., MTC, the amplitude when the person could not tolerate any further increase in stimulation intensity) for each combination of electrode–stimulator in a randomized order. We then used the Biodex dynamometer (System 3, Biodex Medical Systems, USA) to measure the muscle torque generated during an isometric elbow flexion. Each participant was seated in the Biodex dynamometer’s chair with straps placed across their torso to hold their position in place, their arm on the chair’s armrest in a supine position, and their hand holding the arm attachment’s handle and a self-adhesive bandage wrapped around their hand to hold it in place. The experimental setup can be seen in Fig. 12. Based on each individual’s stimulation thresholds, we determined three different intensity levels to test for each electrode–stimulator combination (low, moderate, and high) based on the following formulas:

$$ {\text{Low = mCT + 0}}{.25}\,{*}\,{\text{(MTC - mCT)}} $$

$$ {\text{Moderate = mCT + 0}}{\text{.50 * (MTC - mCT)}} $$

$$ {\text{High = mCT + 0}}{\text{.75 * (MTC - mCT)}} $$

Before stimulating at the determined intensity levels, participants performed three voluntary contractions using their maximum effort and held each for five seconds. We used the average of the three maximum voluntary contractions to normalize the stimulation-induced torque recordings for each individual. The testing order of the electrode type and stimulator was again randomized for each individual. It should be noted that we tested each electrode type with all three stimulators before changing the electrode type. With each combination, we stimulated once to the maximum tolerated level established previously, and then three times at each intensity level (low, moderate, and high) in a randomized order. Stimulation was held for five seconds after reaching the desired intensity level. After each stimulation round, participants rated their comfort level using a visual analog scale from 0 to 10 displayed in front of them where 0 was very comfortable and 10 was very uncomfortable, shown in Fig. 12. After all rounds of stimulation with an electrode–stimulator combination, we also asked ten of the participants to answer an adapted version of the short-form McGill Pain Questionnaire used for electrical stimulation and shown in Additional file 1: Fig. S5, where there were fourteen sensation options (‘throbbing’, ‘shooting’, ‘pricking’, ‘stabbing’, ‘sharp’, ‘cramping’, ‘gnawing’, ‘pulling’, ‘hot-burning’, ‘tingling’, ‘stinging’, ‘aching’, ‘tender,’ and ‘splitting (cutting)’). Each sensation could be rated on a level from 0 to 3 (0 = none, 1 = mild, 2 = moderate, and 3 = severe), depending on how much they felt each one [14, 23, 24].

Torque data were recorded from the analog outputs of the Biodex using a data acquisition system software (LabChart, PowerLab, AD Instruments, USA) at a sampling rate of 1 kHz. All data was then analyzed using MATLAB (v.2021a, Mathworks, USA). For the statistical analysis of the generated torque and comfort ratings, we used a Kruskal–Wallis test.

### Supplementary Information

**Additional file 1: Figure S1.** Each individual participant’s comfort ratings for each combination of electrode–stimulator. 0= most comfortable and 10= most uncomfortable. The darker colors indicate higher comfort. **Figure S2.** Each individual participant’s normalized torques for each combination of electrode–stimulator. The darker colors indicate higher torque. **Figure S3.** Each individual participant’s reported sensations for each combination of electrode–stimulator. Darker colors indicate the sensation was more highly perceived. **Figure S4.** 3D-plots showing average amount of torque generated vs. comfort rating vs. intensity of stimulation used at each intensity level. **Figure S5.** Questionnaire used to describe sensations felt during stimulation.

## Data Availability

The data sets used and/or analysed during the current study are available from the corresponding author upon reasonable request.
