# Supplementary material for: A dry polymer nanocomposite transcutaneous electrode for functional electrical stimulation
Source: Biomed Eng Online. 2024 Jan 26;23:10. doi: 10.1186/s12938-024-01200-8 (PMC10811815; doi:10.1186/s12938-024-01200-8)
Supplement: Supplementary file 1 — Additional file 1: Figure S1. Each individual participant’s comfort ratings for each combination of electrode–stimulator. 0= most comfortable and 10= most uncomfortable. The darker colors indicate higher comfort. Figure S2. Each individual participant’s normalized torques for each combination of electrode–stimulator. The darker colors indicate higher torque. Figure S3. Each individual participant’s reported sensations for each combination of electrode–stimulator. Darker colors indicate the sensation was more highly perceived. Figure S4. 3D-plots showing average amount of torque generated vs. comfort rating vs. intensity of stimulation used at each intensity level. Figure S5. Questionnaire used to describe sensations felt during stimulation. [file 12938_2024_1200_MOESM1_ESM.docx]

**Additional file Information**

**A Dry Polymer Nanocomposite Transcutaneous Electrode for Functional Electrical Stimulation**

Melissa Marquez-Chin, Zia Saadatnia, Yu-Chen Sun, Hani E. Naguib, Milos R. Popovic


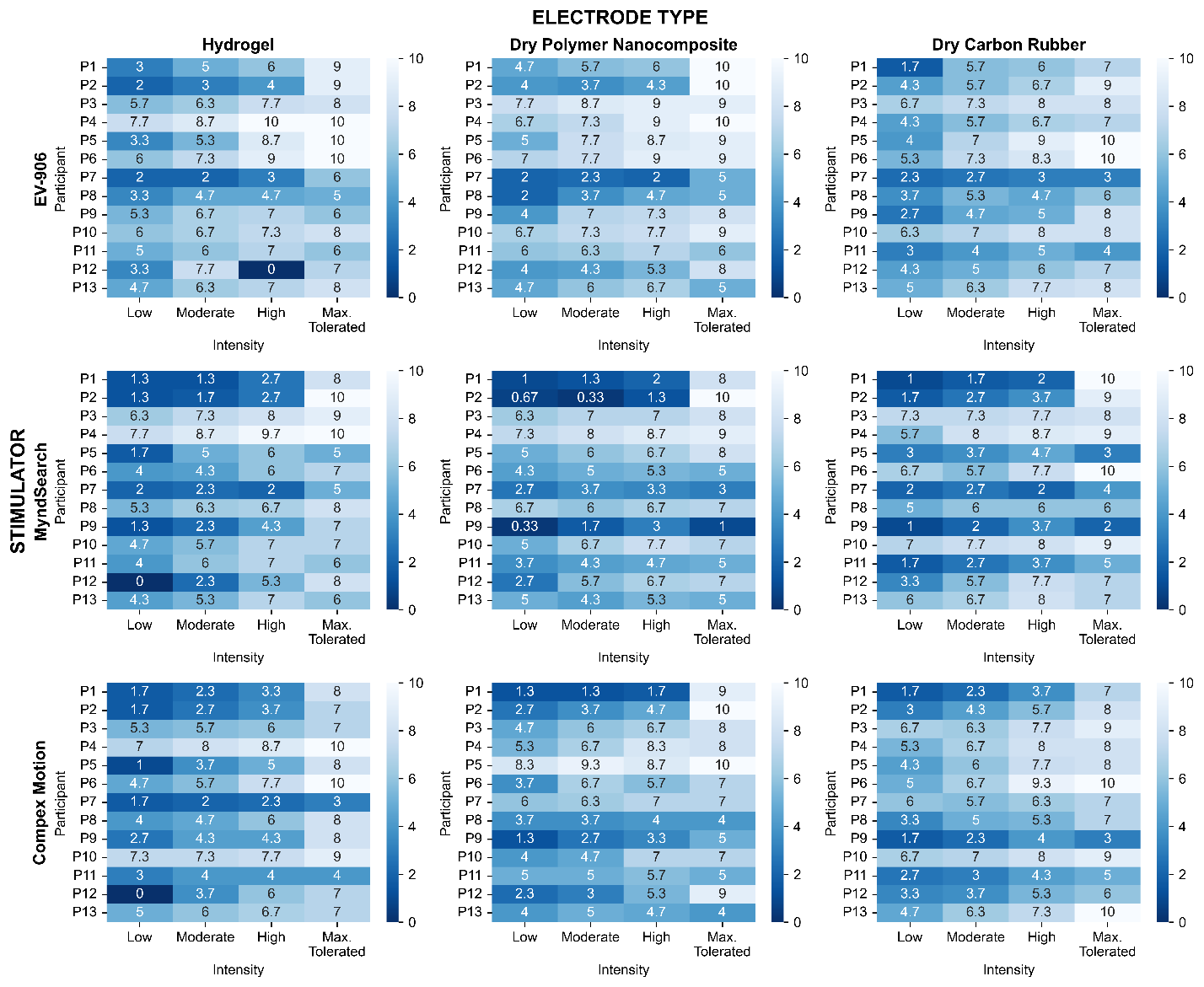


Additional file Figure S1. Each individual participant’s comfort ratings for each combination of electrode-stimulator. 0= most comfortable and 10= most uncomfortable. The darker colors indicate higher comfort.


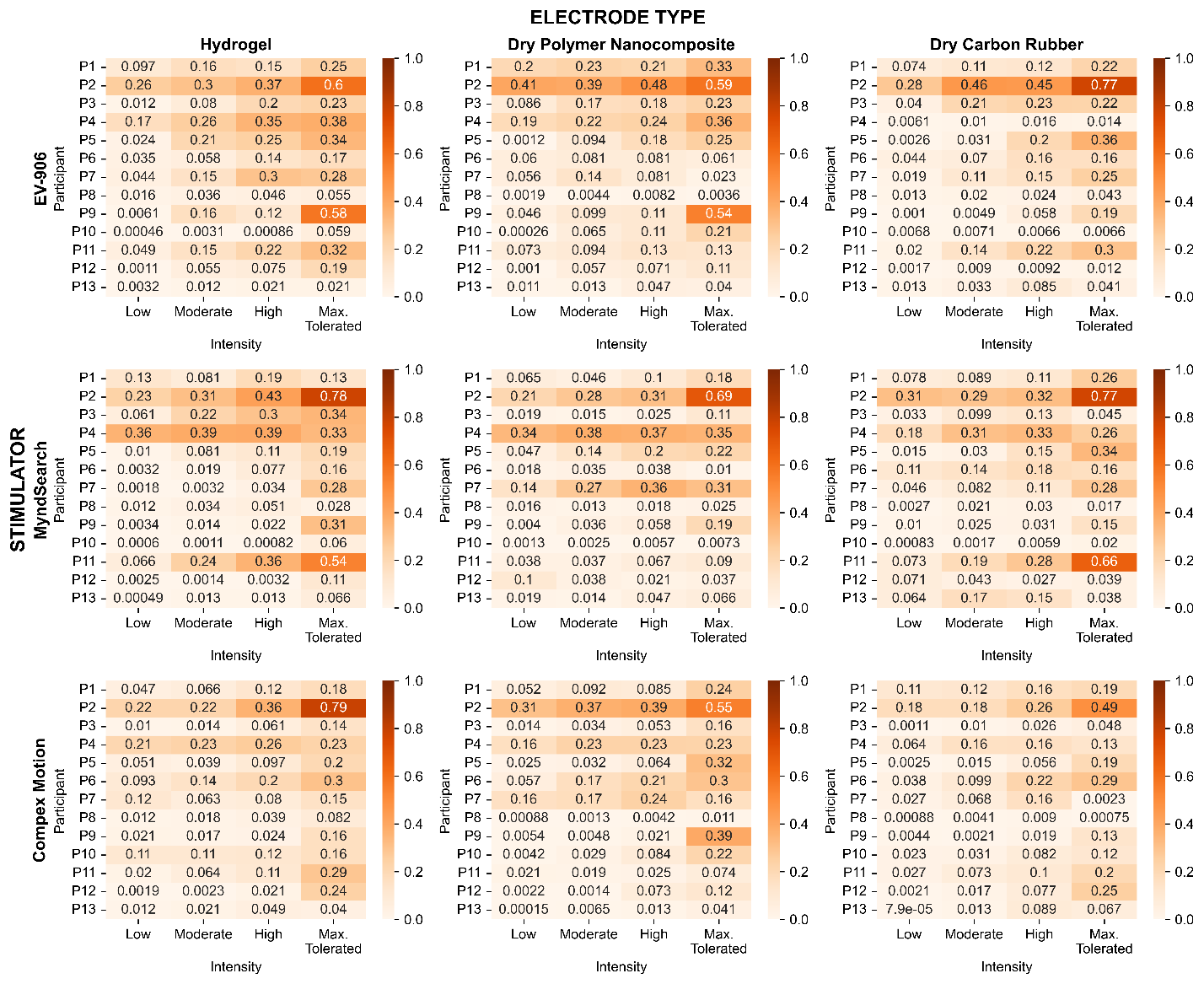


Additional file Figure S2. Each individual participant’s normalized torques for each combination of electrode-stimulator. The darker colors indicate higher torque.


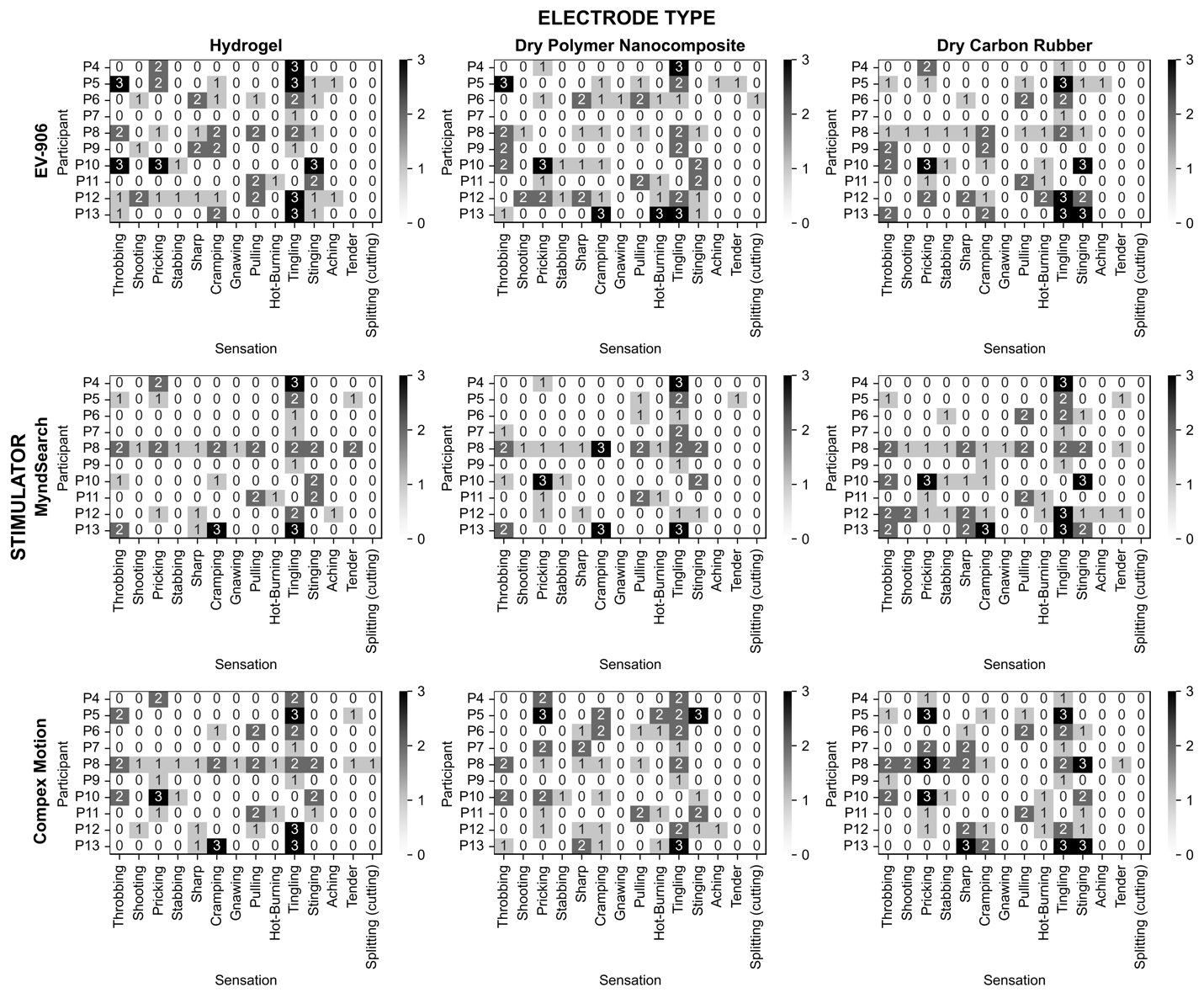


Additional file Figure S3. Each individual participant’s reported sensations for each combination of electrode-stimulator. Darker colors indicate the sensation was more highly perceived.


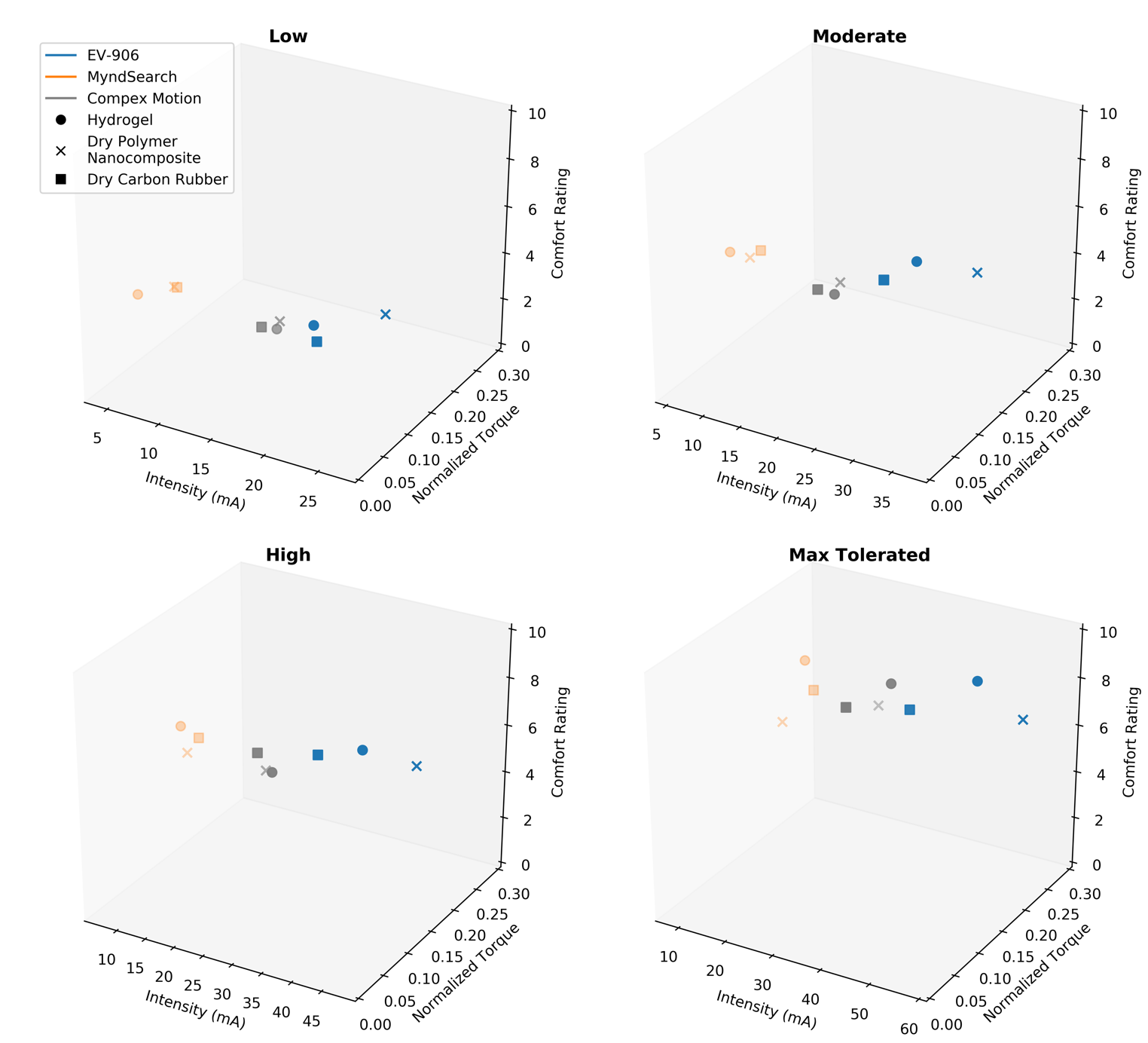


Additional file Figure S4. 3D-plots showing average amount of torque generated vs. comfort rating vs. intensity of stimulation used at each intensity level

| **Sensation** | **None**  **0** | **Mild**  **1** | **Moderate**  **2** | **Severe**  **3** |
| --- | --- | --- | --- | --- |
| Throbbing |  |  |  |  |
| Shooting |  |  |  |  |
| Pricking |  |  |  |  |
| Stabbing |  |  |  |  |
| Sharp |  |  |  |  |
| Cramping |  |  |  |  |
| Gnawing |  |  |  |  |
| Pulling |  |  |  |  |
| Hot-Burning |  |  |  |  |
| Tingling |  |  |  |  |
| Stinging |  |  |  |  |
| Aching |  |  |  |  |
| Tender |  |  |  |  |
| Splitting (cutting) |  |  |  |  |

Additional file Figure S5. Questionnaire used to describe sensations felt during stimulation.
